# Supplementary material for: Distinct Effector Programs of Brain-Homing CD8+ T Cells in Multiple Sclerosis
Source: Cells. 2022 May 13;11(10):1634. doi: 10.3390/cells11101634 (PMC9139595; doi:10.3390/cells11101634)
Supplement: Supplementary file 1 [file cells-11-01634-s001.zip › Cells_Figure S1_revised.pdf]

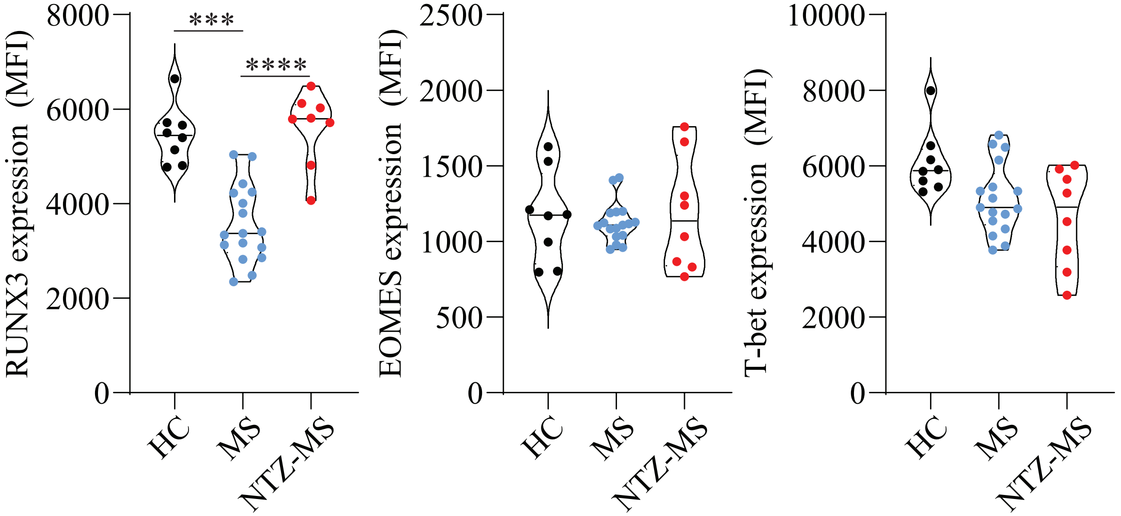

Supplementary Figure S1. RUNX3, EOMES and T-bet expression levels. RUNX3, EOMES and T-bet expression (median fluorescence intensity: MFI) by the RUNX3^{+}, EOMES^{+} and T-bet^{+} CD8+ memory T-cell pool respectively (HC, n = 8; MS, n =18 and NTZ-MS, n = 8). Data were compared using Kruskal-Wallis tests with FDR-BKY corrections. ***p < 0.001 and ****p < 0.0001. "HC" = healthy controls, "MS" = treatment-naive MS patients, "NTZ-MS" = MS patients that clinically responded to natalizumab treatment for 18 months.
