# Supplementary material for: Distinct Effector Programs of Brain-Homing CD8+ T Cells in Multiple Sclerosis
Source: Cells. 2022 May 13;11(10):1634. doi: 10.3390/cells11101634 (PMC9139595; doi:10.3390/cells11101634)
Supplement: Supplementary file 1 [file cells-11-01634-s001.zip › Cells_Figure S2_revised.pdf]

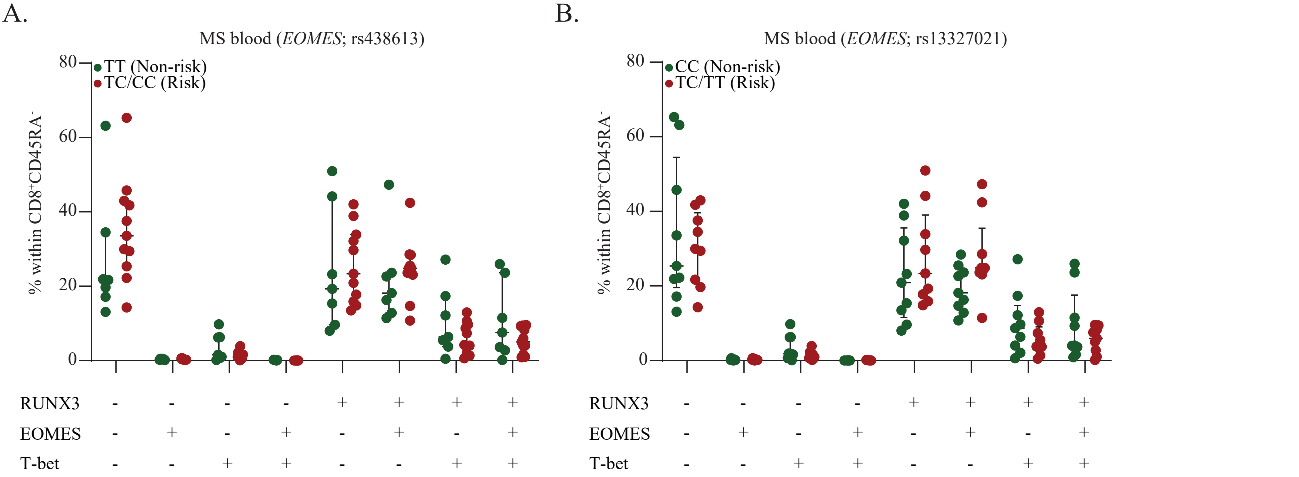

Supplementary Figure S2. The impact of rs438613 and rs13327021 on RUNX3, EOMES and T-bet expression patterns. (**A**) RUNX3, EOMES and T-bet (co)expression by the blood $CD8^+$ memory T-cell pool of MS patients that did (n = 11, TC/CC) or did not (n = 7, TT) carry the rs438613 risk-allele. (**B**) RUNX3, EOMES and T-bet (co)expression by the blood $CD8^+$ memory T-cell pool of MS patients that did (n = 9, TC/TT) or did not (n = 9, CC) carry the rs13327021 risk-allele. Data were compared using two-way ANOVA tests with FDR-BKY correction. “MS” = treatment-naive multiple sclerosis patients.
