# Supplementary material for: Distinct Effector Programs of Brain-Homing CD8+ T Cells in Multiple Sclerosis
Source: Cells. 2022 May 13;11(10):1634. doi: 10.3390/cells11101634 (PMC9139595; doi:10.3390/cells11101634)
Supplement: Supplementary file 1 [file cells-11-01634-s001.zip › Cells_Figure S4_revised.pdf]

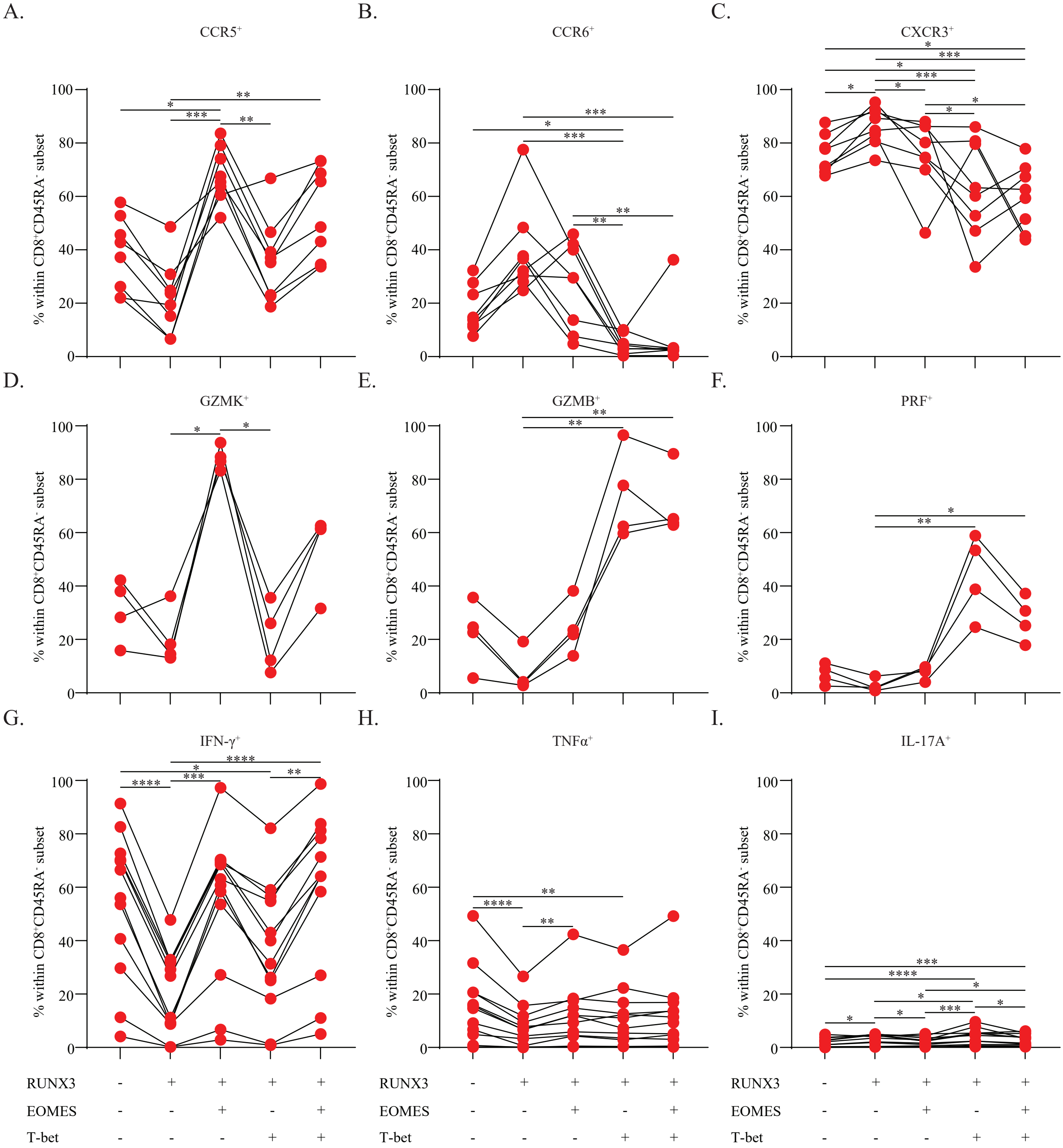

Supplementary Figure S4. The impact of RUNX3, EOMES and T-bet co(expression) on brain-homing, cytotoxic marker and cytokine expression by $CD8^+$ memory T cells from natalizumab-treated multiple sclerosis patients. CCR5 (**A**), CCR6 (**B**), CXCR3 (**C**), GZMK (**D**), GZMB (**E**), PRF (**F**), IFN-γ (**G**), TNFα (**H**), IL-17A (**I**) expression by the $CD8^+$ memory T-cell pool (n = 8 CCRs, n = 4 GZMs and n = 12 cytokines) with RUNX3, EOMES and T-bet (co)expression. Lines represent paired measurements for the same donor. Data were compared using Friedman tests with FDR-BKY correction. *$p < 0.05$, **$p < 0.01$, ***$p < 0.001$ and ****$p < 0.0001$.
