# Supplementary material for: Distinct Effector Programs of Brain-Homing CD8+ T Cells in Multiple Sclerosis
Source: Cells. 2022 May 13;11(10):1634. doi: 10.3390/cells11101634 (PMC9139595; doi:10.3390/cells11101634)
Supplement: Supplementary file 1 [file cells-11-01634-s001.zip › Cells_Figure S5_revised.pdf]

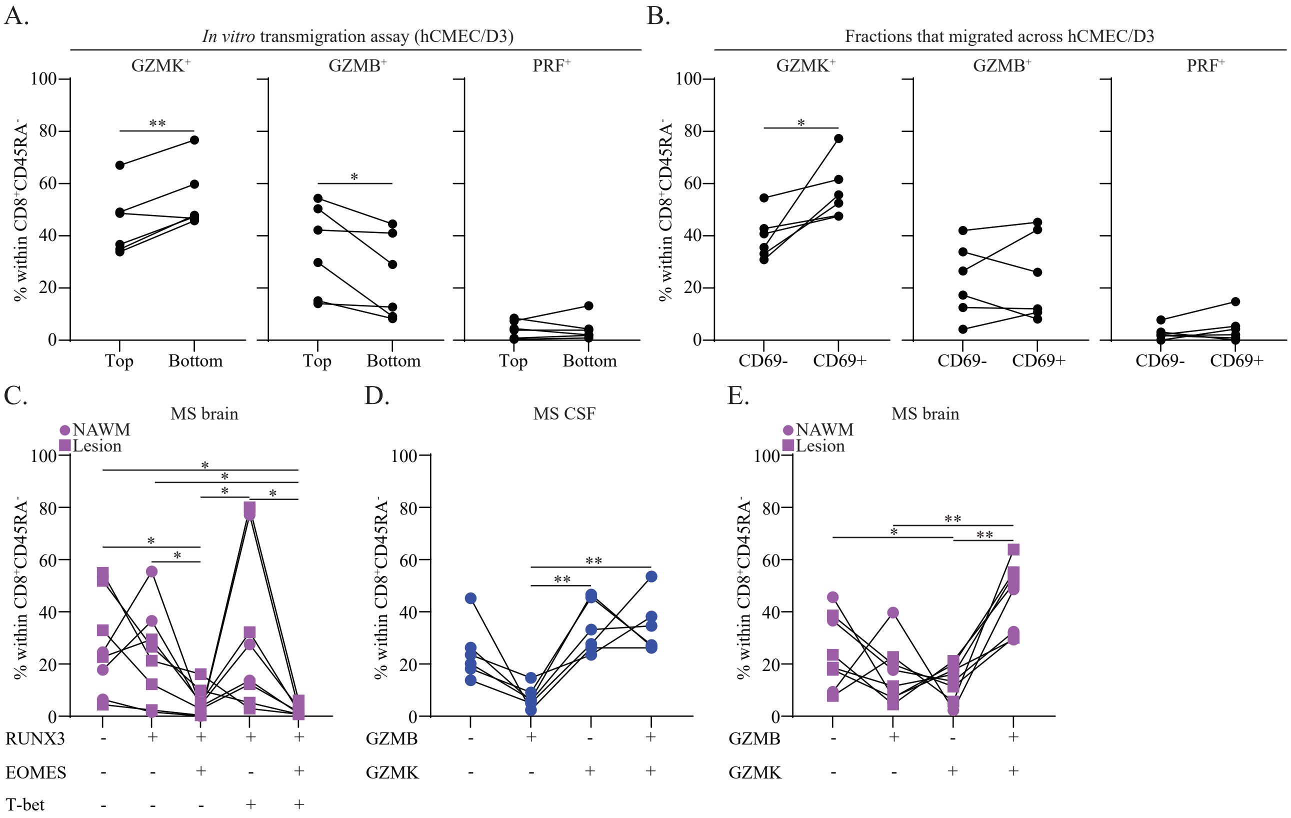

Supplementary Figure S5. Cytotoxic potential of transmigrating CD8$^+$ memory T cells in multiple sclerosis CSF and brain tissue. (**A**) GZMK, GZMB and PRF expression by CD8$^+$ memory T cells of healthy controls (n = 6) that did not migrate (top) or migrated (bottom) across hCMEC/D3 *in vitro*. (**B**) GZMK, GZMB and PRF expression by paired CD69$^-$ versus CD69$^+$ CD8$^+$ memory T cells of healthy controls (n = 6) that migrated across hCMEC/D3 *in vitro*. (**C**) RUNX3, EOMES and T-bet (co)expression by the CD8$^+$ memory T-cell pool in late-stage postmortem multiple sclerosis brain tissue (n = 8 of 4 brain donors). (**D**) GZMB and GZMK (co)expression by the CD8$^+$ memory T-cell pool in early multiple sclerosis CSF (n = 7) and late-stage postmortem multiple sclerosis brain tissue (n = 8 of 4 brain donors). Data were compared using paired t tests (**A**,**B**) or Friedman tests with FDR-BKY correction. “NAWM” = normal appearing white matter, “Lesion” = white matter brain lesion. $*p < 0.05$, $**p < 0.01$.
