# Supplementary material for: Distinct Effector Programs of Brain-Homing CD8+ T Cells in Multiple Sclerosis
Source: Cells. 2022 May 13;11(10):1634. doi: 10.3390/cells11101634 (PMC9139595; doi:10.3390/cells11101634)
Supplement: Supplementary file 1 [file cells-11-01634-s001.zip › Cells_Table S1.pdf]

Table S1: Used antibodies for FACS

Marker	Clone	Fluorescent label	Company
CCR5	J418F1	BV711	Biolegend
CCR6	G034E3	BV421	Biolegend
CD20	2H7	BV421	Biolegend
CD3	SK7	BV786	BD Biosciences
CD3	SK7	AF700	Biolegend
CD4	OKT4	BV510	Biolegend
CD4	OKT4	BV605	Biolegend
CD45RA	HI100	APC-H7	BD Biosciences
CD45RA	HI100	APC Fire 750	Biolegend
CD45RO	UCHL1	BV650	Biolegend
CD69	FN50	BV711	Biolegend
CD69	FN50	BV650	BD Biosciences
CD8	SK1	PerCP-Cy5.5	BD Biosciences
CD8	SK1	AF700	Biolegend
CD8	SK1	FITC	BD Biosciences
CXCR3	G025H7	PE-CF595	Biolegend
EOMES	WD1928	PE-Cy7	Thermo Fisher Scientific
Granzyme B	GB11	PE-CF594	BD Biosciences
Granzyme K	GM26E7	FITC	Biolegend
IFN- γ	4S.B3	BV711	BD Biosciences
IL-17	BL168	APC	BD Biosciences
Perforin	Delta G9 (dG9)	PerCP-eFluor710	Thermo Fisher Scientific
RUNX3	R3-5G4	PE	BD Biosciences
RUNX3	R3-5G4	BV421	BD Biosciences
T-bet	4B10	APC	Biolegend
